# Work stress, life stress, and smoking among rural–urban migrant workers in China

**DOI:** 10.1186/1471-2458-12-979

**Published:** 2012-11-14

**Authors:** Xiaobo Cui, Ian RH Rockett, Tingzhong Yang, Ruoxiang Cao

**Affiliations:** 1 Department of Health Education, Capital Medical University, Beijing, China; 2 Injury Control Research Center and Department of Epidemiology, School of Public Health, West Virginia University, Morgantown, WV, 26506-9190, USA; 3 Center for Tobacco Control Research, Zhejiang University School of Medicine, Yuhangtang Road, Hangzhou 310058, PR China; 4 Institute of Health Education, Beijing Center for Disease Control and Prevention, Beijing, China

**Keywords:** Smoking, Work stress, Life stress, Rural–urban migrant workers

## Abstract

**Background:**

Stimulated by rapid modernization and industrialization, there is massive rural–urban migration in China. The migrants are highly susceptible to smoking and mental health problems. This study examined the association between both perceived work stress and perceived life stress with smoking behavior among this group during the period of migration.

**Methods:**

Participants (n = 1,595) were identified through stratified, multi-stage, systematic sampling. Smoking status separated non-smokers from daily and occasional smokers, and migration history, work stress, and life stress were also measured. Analyses were conducted using the Chi-square test and multiple logistic regression. Two models were utilized. The first was the full model that comprised sociodemographic and migration-related characteristics, as well as the two stress variables. In addressing potential overlap between life and work stress, the second model eliminated one of the two stress variables as appropriate.

**Results:**

Overall smoking prevalence was 64.9% (95% CI: 62.4-67.2%). In the regression analysis, under the full model, migrants with high perceived life stress showed a 45% excess likelihood to be current smokers relative to low-stress counterparts (OR: 1.45; 95% CI: 1.05 – 2.06). Applying the second model, which excluded the life stress variable, migrants with high perceived work stress had a 75% excess likelihood to be current smokers relative to opposites (OR: 1.75; 95% CI: 1.26–2.45).

**Conclusions:**

Rural–urban migrant workers manifested a high prevalence of both life stress and work stress. While both forms of stress showed associations with current smoking, life stress appeared to outweigh the impact of work stress. Our findings could inform the design of tobacco control programs that would target Chinese rural–urban migrant workers as a special population.

## Background

Every year the tobacco-smoking epidemic kills approximately 5.4 million people across the globe. Unchecked, this death toll will exceed 8 million annually by 2030. More than 80% of these deaths will occur in less-developed countries, with the epidemic striking hardest in the rapidly growing economies [1]. China leads the world in tobacco consumption and smoking-related deaths. A recent survey shows that 53% of Chinese men, 2% of the women, and 28% of the overall population (301 million adults) currently smoke tobacco products [2]. Simultaneously, 70% of nonsmoking adults are exposed to secondhand smoke in a typical week.

China is largely agrarian, and farmers comprise more than three-quarters of its total population [3]. Massive rural–urban migration has been stimulated by the rapid modernization and industrialization that is transforming China. Migrant numbers increased from 50 million in 1990 to 121 million in 2000 [4]. Their projected figure for 2010 is 160 million, which would represent approximately 25% of the Chinese working population [5]. Differing from migrants in the traditional sense, rural–urban migrants form a special and vulnerable population group in China. The term “rural–urban migrants,” as used here, refers to individuals who move from rural to urban areas in search of employment and higher living standards, without first establishing permanent urban residence [4].

Rural–urban migrants comprise a distinct and underprivileged group in urban China. Their employment opportunities are severely restricted, and they are denied certain rights, including open employment opportunities, free education, and access to social welfare and to public establishments. Typically, they take only jobs which city residents reject, such as handling of corpses, sewage, chemical waste, and construction work. They live in overcrowded accommodation with poor sanitation, while separated from wives and family. In short, their urban experience appears conducive to high levels of stress [6-8].

Both observational and laboratory studies indicated that stress induces cigarette smoking [9-13]. Many researchers have shown that maladaptation to urban life is probably a key determinant of smoking behavior [6,9,14-18]. Several studies suggest that Chinese rural–urban migrants have an excess smoking prevalence, and that their smoking behavior is linked to unstable living and working conditions [6,16,18,19]. Thus, high stress levels associated with life and work may increase the likelihood of these migrants consuming tobacco products [14,18,19]. Addressing a gap in the literature, this study examined the association between both work stress and life stress, which Chinese rural–urban migrants experience during their transition to urban life, and their smoking behavior. Findings could inform health policy, and help shape the design and implementation of effective tobacco control strategies among this highly vulnerable Chinese subpopulation.

## Methods

### Study design and sampling

This study employed a multi-stage systematic sampling procedure to recruit participants. In Stage 1, two cities, Hangzhou and Guangzhou, were selected from the delta areas of the Changjiang (Yangtze) River in eastern China and the Zhujiang River in southern China, respectively. These two cities were selected because of their relatively open migration policies, and the fact that their economies are among the more developed in China. As a consequence, both cities attract a large number of migrants from rural areas. Guangzhou, the capital of Guangdong Province, had a population of 9.9 million, with an estimated 3.1 million migrants, of whom 1.7 million were men [5]. Hangzhou, the capital of Zhejiang Province, had a resident population of 6.2 million, with an estimated 2.0 million migrants. Approximately 1.2 million of these migrants were male [5]. In Stage 2 of the sampling process, we selected two residential districts with a high density of rural–urban migrants in each of the two study cities, and then randomly sampled half of subdistricts in each district. In Stage 3, worksites were then used as the sampling units. Six categories of worksites were utilized to help ensure diversity among participants. They were construction, machinery and transportation, spin electronics, family services, business, and residual miscellaneous enterprises [6,20]. We then randomly sampled 10% of each of these six worksite categories, which comprised 15, 10, 4, 4, 17, and 25 separate sites, respectively. In Stage 4, the final stage of the survey, we selected, as eligible participants from each district cluster of worksite types, all rural–urban migrant workers who were ages 18 years or older, held rural “hukou,” (i.e., were registered permanent residents in a rural area), and had resided in a destination city for at least six months. Participants numbered about 800 in each city [6,20]. They were distributed across each type of worksite, in proportions corresponding approximately to their estimated overall distribution across each district cluster of worksites.

### Procedures

Employers at the selected worksites were contacted for permission to conduct the survey on their premises. Once permission was obtained, the researchers contacted group “leaders” at each worksite to request them to encourage their workers to participate in the study.

All participants were provided with complete information about the study, and were required to give their verbal consent prior to their participation. Questionnaires were then completed individually and confidentially in an isolated room or other quiet place. When the survey was conducted at a construction site, interviewing occurred in a relatively remote area or an otherwise “quiet spot.” Investigators were available to address participant queries. Assistance was provided to any participants having difficulty in completing the questionnaire, owing to limited education or low reading level. Investigators checked returned questionnaires for completeness. Participants were requested to resolve omissions, as appropriate, and given a token of appreciation (toothbrush and toothpaste, value US$ 0.50) following submission of a completed questionnaire. We contacted a total of 1,673 male migrant workers as eligible participants; 36 could not participate for unknown reasons, and 42 questionnaires were incomplete. Thus, the participation rate was 95% (n =1,595). The study protocol was approved by the Ethics Committee at the Zhejiang University Medical Center.

### Measures

Variables were organized within the questionnaire under the following categories:

Sociodemographics: age, educational attainment, marital status, region of origin, city of residence, occupation, and annual household income.

Migration characteristics: length of stay in city each year (<5 months/5-8 months/≥ = 9 months); period of living with wife during the migration period for married participants (<4 months/4-5 months/≥6 months); and number of proximal friends during the migration period (<4 friends/4-5 friends/≥6 friends).

Perceived life stress questions covered 10 items: (1) instability of living and employment, (2) perceived discrimination, (3) poor living conditions, (4) poor food quality and nutrition, (5) lack of entertainment after work, (6) sexual oppression, (7) poor health (self or family members), (8) familial financial difficulty, (9) obstacles to children’s education, and (10) unhappy marriage [20].

Perceived work stress questions covered 7 items: (1) long working hours and excessive workload, (2) poor relationship with fellow workers, (3) excessively low salary, (4) poor relationship with boss, (5) delay in receipt of payment of wage, (6) poor working conditions, and (7) job insecurity [20].

All items pertaining to the perceived life and work stress measures were rated on a five-point scale: feeling “no stress” (0); “little stress” (1); “some stress” (2); “considerable stress” (3); and “excessive stress” (4). Inapplicable items were assigned a score of zero. A total stress score was obtained by summing up the scores for each item; the higher the total score, the greater the perceived level of stress. Consistent with prior practice, scores exceeding two on each item indicated “high stress” [20]. We calculated total stress scores for both life and work by cumulating scores for each constituent item. High life stress was indicated by a score of 20 or more, and high work stress by a score of 14 or more.

The life and work stress questions utilized for this study were formulated by Yang and colleagues, and the associated stress measures adopted for this study have been used extensively in Chinese research [6,20-22]. Resulting stress scores manifest acceptable validity given strong associations with mental health assessment [20]. They also attain highly reliability in this study (Cronbach alpha values for life stress and work stress scores were 0.83 and 0.85, respectively).

Smoking status: smoking was measured through self-reported use of cigarettes at time of survey. A current smoker was defined as someone who smoked, and comprised both daily and occasional smokers (persons who smoked on some days) [2,6].

### Data analysis

Chi-square tests were used to compare proportions of smokers between groups. Multiple logistic regression analyses were conducted to evaluate respective associations between smoking behavior and geographic, sociodemographic, migratory, and stress characteristics of study participants. For our logistic regression analysis, the dependent variable was smoking status, operationa-lized as a binary response: smokers (self-reported current smoker = 0) and nonsmokers (self-reported non-smoker = 1). In the logistic regression model, we incorporated as independent variables those variables showing statistical significance at 0.025 or less in Chi-square tests. The reference groups in the regression analyses are shown in Table 1. Adjusted odds ratios were estimated, and presented with their associated 95% confidence intervals. The odds ratio represented the likelihood of being a current smoker. P values of 0.05 or less (2-tailed) were considered statistically significant.

**Table 1:** Multiple logistic regression results for sociodemographic, migration, and stress characteristics associated with smoking

	**Model 1**	**Model 2 (*omits life tress*)**
**Age (years)**	OR (95% CI)	OR (95% CI)
<20	Reference	Reference
20-24	1.35 (0.74-2.46)	1.33 (0.74-2.42)
25-29	1.60 (0.87-2.92)	1.60 (0.88-2.94)
30-34	2.18 (1.18-4.04)**	2.22 (1.20-4.12)**
35-39	2.36 (1.16-4.81)**	2.38 (1.17-4.85)**
40+	2.36 (1.171-4.92)**	2.40 (1.16-4.93)**
**Occupation**		
construction	Reference	Reference
machinery and transportation	0.38 (0.27-0.54)**	0.37 (0.26-0.52)**
textiles, electronics, and service	0.55 (0.37-0.82)**	0.52 (0.35-0.77)**
business	0.56 (0.33-0.990)	0.56 (0.32-0.97)*
other	0.20 (0.07-0.58)**	0.18 (0.06-0.53)**
**Length of stay in city each year (months)**		
<5	Reference	Reference
5-8	2.77 (1.43-2.03)**	1.74 (1.24-2.43)**
≥9	1.41 (0.98-1.96)	1.35 (0.96-1.91)
**Life stress**		
low	Reference	
high	1.45 (1.05-2.06)*	
**Work stress**	Not significant	
low		Reference
high		1.75 (1.26-2.45)**

* Significant at p ≤ 0.05.

**Significant at p ≤ 0.01.

We allowed for two models to be used for the regression analysis, since work stress and life stress could be similar concepts. Thus, these two stress variables might overlap when included in the same equation. The first model, our full model, incorporated all of the sociodemographic, migratory, and stress variables. If only one of the two stress variables showed a significant association with current smoking status, under Model 1, we then ran Model 2 in order to be able to evaluate whether the second stress variable was significant in the absence of the first.

We utilized a design weight in our analyses, based on the random selection steps in the multistage sampling design. The selection probability for an eligible individual was calculated as the product of the selection probability of the worksites and the selection probability of migrants at each worksite type. The sampling base weight for an eligible individual was the inverse of the final selection probability, and was assigned to each participant [2]. We conducted our regression analyses using the Complex Samples procedure in SPSS version 17.

## Results

Of the 1,595 participants, 764 were from Guangzhou and 831were from Hangzhou. Their mean age was 29.9 years (SD = 7.9). Their educational attainment distributed as follows: 10% had an elementary school or less education, 51% junior high school, 27% high school, and 12% college or higher. The majority (66%) of participants were married. Those who had never married, or who were divorced or widowed, comprised 32% and 2% of participants, respectively. Forty-seven percent worked in construction, 26% in machinery and transport operations, 16% in textiles and electronics, 7% in business, 2% in services, and 2% in other occupations.

In our sample, the life stressors and work stressors tapped through the survey questionnaire showed satisfactory reliability; respective Cronbach alpha values were 0.83 and 0.85. The mean score for perceived stress from daily life was 2.40 (SD = 0.83, 95% CI: 2.36–2.45). The mean score for perceived stress from work was 2.43 (SD = 0.900, 95% CI: 2.38–2.47). Nineteen percent (95% CI: 17.7–21.4%) of participants were categorized as having high life stress, and 22% (95% CI: 19.6–23.6%) as having high work stress.

Of the 1,595 migrant worker participants in this study, 1,030 were current smokers. Smoking prevalence was 64.9% (95% CI: 62.4-67.2%). Table 2 shows that prevalence varied by age, education, marital status, region of origin, city residence, occupation, length of stay in city each year, number of proximal friends in the migration period, and presence of perceived life stress and work stress.

**Table 2:** Smoking prevalence by sociodemographic, migration, and stress characteristics

**Group**	**N**	**Number of smokers**	**%**	**X**^**2**^	**p**
**City worked**				0.52	0.469
Guangzhou	764	528	63.4		
Hangzhou	831	502	65.9		
**Age (years)**				20.47	0.001
<20	96	49	52.0		
20-24	407	236	58.5		
25-29	408	257	62.6		
30-34	366	253	69.8		
35-39	168	122	74.4		
40+	150	113	74.3		
**Education**				7.68	0.053
elementary school or less	177	132	75.3		
junior high school	831	522	62.4		
high school	413	271	66.9		
college or more	174	105	62.2		
**Region of origin** (based on Chinese administrative regions)				9.96	0.208
North	98	59	60.9		
Southwest	363	225	62.1		
South	376	254	68.2		
Middle East	269	186	70.2		
Southeast	489	306	61.9		
**Marital status**				12.70	0.002
never married	504	285	57.2		
married	1051	719	68.6		
widowed/divorced	40	26	64.2		
**Occupation**				38.24	0.001
construction	754	574	72.4		
machinery and transportation	402	220	53.4		
textiles, electronics, and services	288	172	61.1		
business	126	83	65.2		
other	25	8	36.4		
Annual household income (in Yuan)					
<2000	386	266	60.7	5.09	0.079
2000-3999	482	336	72.1		
4000+	602	428	66.6		
**Length of stay in city each year (months)**				6.78	0.034
<5	339	223	65.7		
5-8	238	169	78.6		
≥9	1018	638	62.8		
**Period of living with wife in migration period** – the married only **(months)**				0.33	0.849
<4	560	396	70.1		
4-5	145	97	58.3		
> = 6	346	226	68.2		
**Number of proximal friends in migration period**				14.38	0.001
<4	551	319	57.6		
4-5	458	321	70.3		
> = 6	586	390	67.6		
**Life stress**				4.28	0.039
low	1284	811	63.4		
high	311	219	71.0		
**Work stress**				5.93	0.015
low	1250	786	63.5		
high	345	244	71.7		

Table 1 shows the multiple logistic regression results. Under the full model (Model 1), which included both stress variables, only perceived life stress showed an association with current smoking. However, when we ran Model 2, which excluded life stress, perceived work stress showed a significant association. In both regression analyses, the adjusted odds ratio increased with age, and was higher for migrants working in construction than for those working in other occupations. Similarly, migrants staying in the city for 5–8 months in a year showed excess smoking prevalence relative to those whose stay was less than 5 months or 9 months or longer.

## Discussion

We estimated current smoking prevalence, and work and life stress status among rural–urban migrants, and identified sociodemographic, migratory, and stress correlates of current smoking. At 64.9% (95% CI: 62.4-67.2%), the current smoking prevalence is significantly higher than the estimate for a national male sample, which was based on data from the Global Adult Tobacco Survey (GATS) (52.1%, 95% CI: 49.7-54.5%)[2]. One of our previous studies showed that smoking prevalence increased among rural–urban migrants after they moved to the city [6]. In our current study, we estimated that 19.5% of rural–urban migrants had high life stress and 21.6% high work stress. We found no previous studies on work and life stress for this population that utilized standard methods of measurement. However, a number of studies showed a high prevalence of stress and mental health problems among Chinese rural–urban migrants [7,8,20,22]. In another of our studies, we found high stress among rural–urban migrants, with the mean CPSS score being 26.4 (95% CI: 26.2–26.60), as compared to 23.8 (95% CI: 23.5–24.1) for male urban residents and 24.8 (95% CI: 24.7–25.0) for rural counterparts [23,24]. Collectively, these findings point to excess prevalence of smoking and stress among rural–urban migrants, and in the process reveal a very important and interconnected social and public health issue.

Addressing a gap in the literature, our exploratory study examines associations between both perceived work stress and life stress with current smoking among the Chinese rural–urban migrant population. Based on running two alternative models, Models 1 and 2, we found that migrants with high work stress had 1.75 times the likelihood of being current smokers as those with low work stress, and the corresponding likelihood for life stress was 1.45 times. These preliminary findings are consistent with those from other studies, which report that high levels of stress from multiple stressors may increase the likelihood of tobacco consumption [6,9-13,16,18,19]. Our findings, in concert with comparative data from previously documented studies, suggest a special need to ameliorate stress among the migrants, since they show both an excess of high stress and smoking behavior relative to urban residents [2,24]. For this reason, the Central government should consider changing the current policy on the household registration system, so as to guarantee the migrants the same legal rights of citizenship at the urban destination as local residents. Only through elimination of this major impediment to equality will there be improvement in the working and living conditions of these migrants.

Our study found that 25% of rural–urban migrants reported that low pay was a source of high work stress, the largest percentage among the items that we employed in measuring that variable. These migrants perform arduous work that yields low economic returns. Indeed, this effort-reward imbalance generates high stress levels, which in turn are conducive to smoking [25]. However, low pay likely induces high work stress, independent of migrant status, and indeed other studies have shown that high effort-low reward relationships promote smoking [19,26].

In the presence of the perceived life stress variable, perceived work stress did not show an association with current smoking among rural–urban migrants (Model 1). It did in its absence (Model 2). This finding implies that there is a major overlap in the two stress concepts, or at least in the way that we operationalized them. Our future research will need to consider stress-smoking relationships in terms of separate and joint contributions from the respective items constituting our perceived stress measures. Analytic longitudinal research will be essential for appropriately evaluating the absolute and relative importance of life stress and work stress as determinants of smoking behavior, within the context of the migration experience.

Our results reinforce observations that stress is a risk factor for smoking [6,9-16]. Several studies suggest that Chinese rural–urban migrants have excess smoking prevalence, and that their smoking behavior is linked to high stress [6,16-18,21]. Our study revisited this question from a broader perspective. Prior studies found that working conditions are associated with smoking among rural–urban migrants, and that high work stress may increase their prevalence of tobacco use [6,17,18,21]. Rural–urban migrants are industrious, have low wages, and the effort-reward imbalance elevates stress and promotes smoking [25].This association has been tested in other populations, with higher effort-lower reward combinations increasing smoking prevalence [19,26]. Several studies noted that rural–urban migrants encounter many daily challenges [7,8,17,20]. These migrants live in overcrowded accommodations with poor sanitation, frequently reside separately from wives and family, have low family incomes, and their children lack basic education. We found that such high life stress adversely impacts their smoking behavior. Previous research has shown that the prevalence of smoking initiation increases with unstable living conditions [6,16,18,21]. In contrast to previous studies, we factored instability of both living and employment into our life-stress component. Collectively, work stress and life stress arise from and exacerbate the challenges faced by rural–urban migrant workers [20]. In running Model 1, we found that life stress suppresses work stress as a correlate of smoking among the migrant workers. This finding fits the reality that, typically, the migration process first means profound separation from family, with concomitant personal problems later becoming compounded by the strain of seeking, obtaining, and retaining city employment. Coming to the fore in this problematic situation is the need for Government to guarantee rights and benefits to this highly vulnerable rural–urban migrant population.

Confirming prior findings [6,16,17], we found that current smoking prevalence increased with age among rural–urban migrants. Migrants working in construction tended to have a higher smoking prevalence than those engaged in other occupations. Although construction is difficult and hazardous work, wages are very low, and payment is frequently delayed or withheld. This constellation of factors may contribute to excess smoking among such migrant workers [6,17,21]. A potential alternative explanation for their excess smoking may be that employment in construction facilitates on-the-job smoking.

Length of stay in the city each year was positively associated with current smoking. This finding provides additional support for the notion that stress is a by-product of the struggle to adapt to a different existence [6,16,18]. Migrants residing in the city between five and eight months each year were more likely to smoke than those with either shorter or longer duration. This may be the fact that migrants staying in cities for the longest annual duration may be adapting more easily than intermediate-duration counterparts to the urban environment, and also coping better with concomitant stress. Salient to these empirical questions, some studies found that the prevalence of initiation of daily smoking increased with the number of cities in which the migrants had lived [6,16], a finding that implicated unstable living and working conditions [6,16,21].

Rural–urban migrants have little education, and they lack stress-management skills and awareness of tobacco control [6,16-18,21]. Government and local health authorities should consider stress management and tobacco control as crucial components of healthcare. These authorities need to develop policies for prevention or amelioration of stress and smoking problems in general, as well as to design and implement interventions that target rural–urban migrant workers in particular. A national imperative is to provide this group with a tailored community-based stress management and tobacco control education curriculum. This curriculum should address such topics as stress risk-factor identification and management, together with other smoking countermeasures.

Our study has several major limitations. Given that sampling of rural–urban migrants is a formidable challenge, we found it infeasible to obtain a true probability sample of the target population. Consequently, we could not capture the total complexity of the rural–urban migrant experience, an issue we plan to revisit in future research. Another limitation was that our cross-sectional study design precluded causal inference. Moreover, the generalizability of results was constrained because our study was confined to male migrant workers, owing to low smoking prevalence among females. To address this gender prevalence gap, future research will likely need to oversample females. A final study limitation, we assessed smoking status through self-report and without biochemical validation. Such assessment may introduce information bias. On the other hand, self-reported data are the conventional product from population-based smoking surveys, including the GATS [2]. We consider that the appropriateness of our data is reinforced by evidence that self-report bias in smoking research is minimal [27,28]. Since smoking represents normative behavior for adults in China, especially with regard to males, any social inhibition of accurate reporting is plausibly but a minor concern.

## Conclusion

This study contributes to the general smoking literature by exploring the association between work and life stress among Chinese male rural–urban migrant workers on the one hand, and their smoking status on the other. While preliminary, our findings support the need for creation and implementation of policies and strategies that address smoking through intervention in both stress and smoking pathways.

## Competing interests

The authors declare that they have no competing interests.

## Authors’ contributions

TY conceived the study design, conceptualized the problem, and supervised data management and analyses. XC and RC conducted the data collection. IR provided technical support for the data analysis, and revised and edited the manuscript. All authors reviewed earlier drafts and approved the final version.

## Pre-publication history

The pre-publication history for this paper can be accessed here:

http://www.biomedcentral.com/1471-2458/12/979/prepub
